# Tourette syndrome research highlights from 2019

**DOI:** 10.12688/f1000research.27374.2

**Published:** 2020-12-04

**Authors:** Andreas Hartmann, Yulia Worbe, Kevin J. Black

**Affiliations:** 1 Department of Neurology, APHP, Paris, Île-de-France, 75013, France; 2 Department of Psychiatry, Neurology, and Radiology,, Washington University School of Medicine, Saint Louis, MO, 63110, USA

**Keywords:** Tics, Tourette syndrome, 2019

## Abstract

This is the sixth yearly article in the Tourette Syndrome Research Highlights series, summarizing research from 2019 relevant to Tourette syndrome and other tic disorders. The highlights from 2020 is being drafted on the Authorea online authoring platform; readers are encouraged to add references or give feedback on our selections comments feature on this page. After the calendar year ends, this article is submitted as the annual update for the Tics collection F1000Research.

## Introduction

This article is meant to disseminate recent scientific progress on Gilles de la Tourette Syndrome (TS).

## Methods

We searched PubMed from time to time during 2019 using the search strategy “(”Tic Disorders“[MeSH] OR Tourette NOT Tourette[AU]) AND 2019[PDAT] NOT 1950:2018[PDAT]”. Colleagues also recommended articles, and we attended selected medical conferences. We selected material for this review subjectively, guided by our judgment of possible future impact on the field.

## Results

### Phenomenology and natural history

Müller-Vahl *et al.* ^1^ present a stimulating argument based on data that demonstrate a severity continuum between chronic tics and TS using a database regrouping 1018 subjects. They thus conclude that TS and chronic tics do not represent distinct diagnostic categories. Accordingly, they suggest the introduction of the the term “tic spectrum disorders” (in analogy to autism spectrum disorders, ASDs) which might present the added benefit of decreased social stigma related to TS.

Martino and Hedderly provide an excellent review on the differences between tics and stereotypies, and their clinical management ^2^

A useful resource is the video atlas of various vocalizations that includes tics and helps with differential diagnosis ^3^.

***Epidemiology*.** Several good epidemiological studies and meta-analyses on the childhood prevalence of TS have been published over the past years, with most figures ranging between 0.5 to 0.8%, but they could be much higher. However, prevalence in adulthood remains unknown. Levine *et al.* analysed three studies (published 1986, 2011 and 2016) involving 2,356,485 participants ^4^. Overall prevalence of TS in adulthood was estimated to be 118 cases of TS per million adults, that is 0.0118%. This appears very low, even factoring in remission in two thirds to three quarters of childhood cases during adulthood (which, in itself, is debatable). Clearly, more research is needed on this important topic, preferably using current DSM-5 criteria.

***Tic suppression*.** Forty-five children with tics starting on average only 3–4 months ago were assessed with clinical and psychological methods and reassessed at the 12-month anniversary of their first tic ^5^. Children who were less able at the first visit to suppress tics while given immediate rewards for 10-second tic-free intervals had worse clinical status (higher YGTSS total tic score) at the follow-up visit six to nine months later. This finding adds to the meager prognostic clues available for Provisional Tic Disorder a simple, clinically relevant test.

***Sensory phenomena and premonitory urges*.** Rae and colleagues provide a very thoroughly discussed computational model of how tics and premonitory sensations may be generated ^6^. The model links premonitory phenomena and tics to a hypothesized overly precise internal estimate of sensory information and predicted movement, and has the key advantage of generating some testable hypotheses.

***Other*.** David Mataix-Cols’ group continue their epidemiological exploration of patients with TS and chronic tic disorders (CTD) using the Swedish National Patient Register. This time, they looked for metabolic and cardiovascular disorders in these patients and find that the risk is doubled compared to the general population, especially with regard to obesity, type 2 diabetes and circulatory system diseases. With regard to co-morbidities, the presence of attention-deficit / hyperactivity disorder (ADHD) significantly increased the risk (however, excluding ADHD does not normalize the risk, still 50% higher than in the general population). Most surprisingly, use of antipsychotic medication for more than one year was associated with a significantly *decreased* risk for metabolic and cardiovascular disorders in patients with TS or CTD. This counterintuitive finding, given antipsychotics’ propensity to induce metabolic syndrome, requires further clarification. For now, the authors speculate that patients with TS or CTD receiving medication benefit from frequent follow ups and better monitoring of their general health. In any case, this is a further demonstration, after papers on suicide and educational attainment in patients with TS or CTD, that chronic tic disorders are far from benign and require correct diagnosis, then regular care and follow up ^7^.

### Etiology

***Genetics*.** 2019 has seen the publication of a variety of studies using whole exome sequencing. Depienne *et al.* ^8^ investigated 120 TS patients and identified disrupting variants of OPRK1, encoding the opioid kappa receptor, in a significant subset of subjects compared to controls. This result points to a role, discussed since the 1980s, of the opioid system as involved in the pathophysiology of TS and also suggest a new potential therapeutic target.

After whole exome sequencing of 100 trios (TS patients and their parents), point mutations in ASH1 Like Histone Lysine Methyltransferase (ASH1L) causing defects in its enzymatic activity were identified as a susceptibility gene for TS ^9^, previously associated with mental retardation and autism. A transgenic mouse line ( *Ash1l* heterozygous mice) indeed displayed tic-like motor and compulsive behaviors, and dopaminergic hyperinnervation was observed in the dorsal striatum, demonstrating good construct validity for this model.

Two more genes, chromodomain helicase DNA binding protein 8 (CHD8) and Signal Peptide, CUB Domain And EGF Like Domain Containing 1 (SCUBE1), were identified by whole exome sequencing in a cohort of 222 OCD parent-child trios, and it was further shown that these genes overlap with genes previously implicated in TS ^10^. Of note, Katayama *et al.* (2016) demonstrated that mice heterozygous for Chd8 mutations manifest ASD-like behavioral characteristics including increased anxiety, repetitive behavior, and altered social behavior ^11^.

Using the Swedish National Registry, it was shown that maternal polycystic ovary syndrome (PCOS), as a model for investigating the role of prenatal androgen exposure, is a risk for TS, ADHD and ASD ^12^. These results support a potential causal influence of prenatal androgen exposure on the development of male-predominant neuropsychiatric disorders in female offspring of women with PCOS.

Still in Sweden, Brander *et al.* ^13^ investigated whether, at the population level, tic-related OCD has a stronger familial load than non-tic-related OCD. They found that the risk of OCD in relatives of individuals with tic-related OCD was considerably greater than the risk of OCD in relatives of individuals with non-tic-related OCD, concluding that tic-related OCD is a particularly familial subtype of OCD. The results have important implications for ongoing gene-searching efforts.

***Environmental risk factors*.** The EMTICS study was a large European multicenter trial investigating, among several subjects, the role of immunology in the etiology of tics, a long-discussed hypothesis in the context of Pediatric Acute-onset Neuropsychiatric Syndrome (PANS) / Pediatric Autoimmune Neuropsychiatric Disorders Associated with Streptococcal Infections (PANDAS). A first paper on neuronal surface proteins on 188 patients with TS failed to confirm a link between pathogenic antibodies and causation of tics ^14^. In line with these findings, Baumgaertel *et al.* ^15^ failed to detect autoantibodies in the CSF of 20 adult patients with TS. However, 20% of these patients had positive oligoclonal bands, an intriguing finding with no clear-cut explanation to date. Also, in the neuroimmunological field, Gilbert provides a thoughtful review of the PANDAS/PANS controversy ^16^.

Chen *et al*. ^17^, using the National Health Insurance Research Database of Taiwan, analyzed 2261 TS patients and 20349 non-TS controls for the risk of traumatic brain injury (TBI). During follow-up, there was a significantly increased risk for TBI in TS patients compared to controls. Classic comorbidities such as ADHD, OCD and depression increased the risk for TBI, whereas the *regular* use of antipsychotic medication decreased it. These findings have important therapeutic implications, as it underlines the need to offer proper and sustained anti-tic treatment in patients, even if these treatments may be sedative, as may be the case for antipsychotics.

### Pathophysiology

Singer and Augustine have published two excellent and exhaustive reviews on the pathophysiology of tics/TS (including controversies) and the relevance for pharmacotherapy ^18, 19^.

***Electrophysiology*.** Loo *et al.* ^20^ performed a 128-channel electroencephalogram (EEG) study on children with TS during an exaggerated blink task and showed overall higher gamma band spectral power and differences in theta, alpha, and beta band power in inferior parietal cortex in TS children compared to controls.

Niccolai *et al.* ^21^ studied motor-related beta oscillations in TS using magnetoencephalography and showed a biphasic increase-decrease pattern of beta oscillations. The decrease of beta oscillations was observed close to tic execution, similarly to what was observed in voluntary actions. The initial increase in beta power positively correlated with premonitory urges. Similarly, Zaparolli *et al.* ^22^ studied the neural activity over the sensorimotor cortex using EEG during a finger movement task in TS and found decreased levels of beta modulation compared to controls in tic-free conditions. However, the abnormal pattern normalized if the patients were actively suppressing tics during the task.

 Zhu *et al.* ^23^ recorded local field potentials in the globus pallidus internus (GPi) and sub-thalamic nucleus (STN) of patients with TS and found that beta and gamma oscillations in the GPi were restored after deep brain stimulation (DBS) of the GPi but not after DBS of the STN, suggesting that these oscillations may play a role in pathophysiology of persistent tics. Another study using microelectrode recordings of the STN during DBS surgery in a single TS patient was able to identify a single unit activity of the STN within the delta band, which was reliably associated to optimal DBS target site for tic control ^24^.

***Neuroimaging studies*.** Ramkiran *et al.* ^25^ used graph theoretical measures applied to resting-state functional magnetic resonance imaging (fMRI) in adults with TS and studied functional properties of different portions of cortico-basal ganglia-cerebellar networks. They showed increased basal ganglia-cortical and thalamo-cortical connectivity but reduced cortico-cerebellar connectivity compared to controls. The authors also reported reductions in serial information transfer within the default mode and the salience functional networks. Altogether, the findings suggested disruption of interoceptive mechanisms and of brain maturation, as well as a shift towards excitatory neurotransmission in TS.

Sigurdson *et al.* ^26^ focussed on cerebellar morphology and structural connectivity (structural co-variance) in TS and found reduced grey matter volumes in part of the cerebellum involved in motor and cognitive information processing compared top controls. The cerebellum also had abnormal structural connectivity with sensori-motor networks and fontal and cingulate cortices. These findings highlight the importance of the cerebellum in tic pathophysiology. The same approach of structural co-variance was used to study the structural underpinnings of premonitory urges with a specific focus on the right insula ^27^. The severity of tics and premonitory urges correlated, respectively, with posterior (representing the current physiological state) and anterior (associated with urges for action) sub-regions of the insula. In addition, these sub-regions of insular cortex were related to different structural networks, suggesting that separate networks support tics and premonitory urges in TS.

In one of the largest to date studies on resting state functional connectivity in adults and children with TS, Neilson *et al.* ^28^ showed that patterns of functional connections alterations were age-dependent: while brain networks in TS children presented features of older age, adult TS brain networks appeared “younger” in comparison to age-matched controls. Overall, these findings underline the differences in TS neurodevelopmental trajectories.

Finally, O’Neil *et al.* wrote a comprehensive review about neuroimaging findings on the role of the cingulate cortex in TS, suggesting that at least six to eight different sub-regions of this cortical area might be implicated in different aspects of TS pathophysiology, and are especially involved with premonitory urges ^29^. Activity in the subgenual and pregenual anterior cingulate as well as in the middle cingulate cortex might represent volitional effort, physical discomfort and emotional distress of premonitory urges; the posterior middle cingulate cortex and dorsal posterior cingulate cortex might play a role in amplification (build-up) of urges.

A positron emission tomography study of 33 adults found that serotonin transporter (SERT) binding in caudate and midbrain was normal in people with tics only or OCD only, but was elevated in people with both tics and OCD ^30^. This result, if replicated, may suggest a nosological distinction between TS with vs. without OCD, which would be surprising from a clinical viewpoint.

***Clinical and neuropsychological studies*.** Recent studies indicate that the coordination of bimanual movements may involve a number of brain areas: primary sensorimotor cortex, supplementary motor area (SMA), premotor cortex, cingulate motor cortex, lateral premotor cortex, basal ganglia, inferior parietal cortex, and the cerebellum (many of which, incidentally, have been reported to be altered with respect to structure and/or function in brain imaging studies of TS). However, it is accepted that interhemispheric transfer is mediated through excitatory and inhibitory transcallosal communications between cortical motor areas and that the corpus callosum therefore plays a major role in the coordination of bimanual movements, particularly asymmetric bimanual movements. A recent study investigated externally paced (cued) and internally paced bimanual tapping in adults with and without TS. Importantly, this study combined behavioral measures of bimanual tapping with MRI-based diffusion tensor imaging and probabi­listic tractography of inter-hemispheric callosal connections between the SMA and the left SMA–putamen fiber tract ^31^ *.* TS patients were significantly less accurate than healthy individuals when asked to maintain a previously copied rhythmic tapping speed at time intervals < 1 Hz ^32^. Unimanual tapping is the condition requiring the greatest level of interhemispheric inhibition. TS patients also showed altered fractional anisotropy in inter­hemispheric (SMA–SMA) and left-sided SMA–putamen fiber tracts. These findings are consistent with compensatory processes linked to self-regulation of motor control that may occur through the plastic rearrangement of interhemispheric and cortical-subcortical WM pathways.

Maigaard and colleagues studied the ability of children with TS to suppress quick but inappropriate rewards ^33^. Not surprisingly, children with ADHD did poorly on this task, but children with TS actually did better than healthy control children. All groups improved their accuracy when a reward was promised for accuracy. One hypothesis to explain these results may be that children with TS have better motor inhibition in certain tasks due to their experience withholding tics in response to premonitory urges due to social pressures. The reward effect may correspond to the known improvement in tic suppression in the presence of immediate rewards ^5, 34^.

### Treatment

***Psychological interventions*.** In a large study of manualized CBT in children with OCD, anxious and depressive symptoms improved substantially and were not linked to improvements in OCD severity ^35^. This result is one more argument in favor of psychotherapy for obsessions and compulsions, which are common in people with tics. A consensus report argues strongly for early intervention in OCD ^36^. Since early-onset OCD is associated with tics ^37^, a similar argument could be made for early intervention in tic disorders, especially since effective behavioral treatments without side effects are available. Studies of whether early intervention changes the course of tic disorders are needed.

One of the most interesting possibilities in delivering behavior therapy for tics has come from the development of internet-based platforms, making these approaches available for a large number of patients, even in remote areas. The BIP-TIC platform, developed in Sweden, allows to use either habit reversal training (HRT), exposure response prevention (ERP) or a mixture of both online with a possible intervention of a therapist by phone or email. A first pilot study on 23 patients has shown encouraging results in a rater-blind parallel group trial (including a 12 month follow up) ^38^. A large (n= +200) UK-based study of ERP using this platform, called ORBIT, will commence shortly ^39^.

Another way to increase the number of patients to be reached by CBT is group therapy. A Danish study, using a combined HRT/ERP approach has demonstrated that it is equally effective in a group as in an individual setting with 27 patients per treatment arm ^40^. This represents a promising and interesting way forward in CBT for tics.

***Medication*.** The American Academy of Neurology (AAN) practice guidelines for TS ^41, 42^ are one of the most important publications in our field for 2019. A detailed analysis goes way beyond the scope of this review but it might be worth noting that the only “high confidence in the evidence” rating was awarded to Comprehensive Behavioral Intervention for Tics (CBIT) and not pharmacological or surgical therapies for tics. This represents a true paradigm shift in the field. Similar conclusions were drawn in another review of evidence-based treatments for TS and CTD ^32^.

In recent years, cannabis and cannabis-derived products are being considered for the treatment of tics – and a variety of other movement disorders. Milosev *et al.* ^43^ present results from a retrospective data analysis and an online survey on the use of cannabis-based medicine for tics and comorbidities in TS. Patients (n= 98 and 40) expressed a preference for medical cannabis (rich in THC) over dronabinol and nabiximols. However, results from large randomized trials are still awaited and will help guide therapeutic decisions. These will also depend, obviously, on the availability of different cannabis-based medications across countries.

***Neurosurgery*.** Blocking tics by behavior therapy or botulinum toxin has been hypothesized to interrupt the sensory-motor feedforward loop likely operating in TS, i.e. premonitory sensations triggering tics which then re-inforce premonitory sensations. Kimura *et al.* ^44^ report on four patients who had undergone thalamic DBS for severe TS. In two, DBS could be completely withdrawn four and seven years after surgery, respectively, without re-increase in tics. This is intriguing and confirms unpublished reports from other centers, including our own (Paris). The authors raise the question of whether some of the tics observed pre-op were functional tics. Alternatively, perhaps some patients with severe tics may need treatment only for several years during development, when tics may have been most severe without treatment. On the topic of functional tics, which are occasionally seen by movement disorder specialists, Ganos *et al.* ^45^ have published a landmark review which should be compulsory reading in the field.

In a 48 month follow-up of a multicentre trial comprising 16 severe TS patients treated with DBS of the anterior pallidum, it was found that 75% of subjects were treatment responders, that YGTSS (-40%) and global functioning scores decreased significantly, and that self-injurious behaviors ceased in all affected (n=7) patients ^46^. Also, no persistent psychiatric or neurological side effects were noted. However, DBS did not lead to overall decrease in medication. Predictors of long-term outcome for DBS in TS are still needed and larger, perhaps international studies will be able to fill that gap. One step towards that was signalled by a report of initial results from an international TS-DBS registry relating DBS active contact location to outcomes ^47^.

***Other treatments*.** Regarding unusual treatment methods for tics, Murakami *et al.* ^48^ describe the use of oral splints in 22 patients with TS. Tic decrease was noted in the vast majority of cases and occurred almost instantaneously. The authors suggest a placebo effect and/or a sensory trick as mechanism of action. The major question here remains how and if such an intervention can work long term and without impairing daily functioning, especially speech.

 A pilot study evaluated the efficacy of a resource activation program as an alternative intervention for children and adolescents (n=24) with tic disorders ^49^. Their preliminary results suggest that after 16 treatment sessions, tics were significantly diminished using the YGTSS and other tic-related measures. Larger cohorts and longer follow-up will hopefully establish whether this approach might become an alternative or adjunct to established CBT approaches for treating tics such as HRT and ERP.

## Conclusions

They are the same as last year (and likely for a while to come) but worth reiterating, and consist of several simple but important questions: Why do tics tend to start at ages 5–10? Why are they more common in boys? Why do they tend to improve during sleep? Why do tics usually improve in early adulthood? How accurately can we predict outcome for an individual patient? Which patients need which treatments? Is secondary prevention possible? Hopefully future studies will address these and other important issues.

## Data availability

No data are associated with this article.
