# Supplementary material for: Tourette syndrome research highlights from 2019
Source: F1000Res. 2020 Dec 4;9:1314. Originally published 2020 Nov 11. [Version 2] doi: 10.12688/f1000research.27374.2 (PMC7808056; doi:10.12688/f1000research.27374.2)
Supplement: Peer review history

# Peer review: Reviewer response for version 2

**DOI:** 10.5256/f1000research.31048.r75789

**Published:** 2021-01-13

**Authors:** Tammy Hedderly

**Affiliations:** 1 Tic and Neurodevelopmental Movements Service (TANDeM), Evelina Children's Hospital, London, UK; 2 GSTT/KCL, London, UK

The authors are now experts in providing a detailed and helpful summary of the research in Tourette. As a practitioner in the field I find these annual updates extremely helpful and know many that share this view. The paper is well referenced and although the summary of each paper is brief it allows for easy access to the relevant papers. Next year I would like to see some of the papers on functional tics included and of course we may expect to see a discussion around the impact of COVID in these challenging times. I strongly support the indexing of this helpful review paper and thank the authors for their helpful summary of this years papers.

Is the review written in accessible language?

Yes

Are all factual statements correct and adequately supported by citations?

Yes

Are the conclusions drawn appropriate in the context of the current research literature?

Yes

Is the topic of the review discussed comprehensively in the context of the current literature?

Yes

Reviewer Expertise:

Lead of Tourette Services-clinical

I confirm that I have read this submission and believe that I have an appropriate level of expertise to confirm that it is of an acceptable scientific standard.

# Peer review: Reviewer response for version 2

**DOI:** 10.5256/f1000research.31048.r75777

**Published:** 2020-12-04

**Authors:** Natalia Szejko

**Affiliations:** 1 Department of Neurology, Medical University of Warsaw, Warsaw, Poland; 2 Department of Neurology, Yale School of Medicine, Yale University, New Haven, CT, USA

Thank you so much for addressing these small issues, now the article reads well, I do not have any further comments.

Is the review written in accessible language?

Partly

Are all factual statements correct and adequately supported by citations?

Yes

Are the conclusions drawn appropriate in the context of the current research literature?

Yes

Is the topic of the review discussed comprehensively in the context of the current literature?

Yes

Reviewer Expertise:

Tourette syndrome, movement disorders, neuropsychiatry, cannabis based medicine in neurology and psychiatry, functional movement disorders

I confirm that I have read this submission and believe that I have an appropriate level of expertise to confirm that it is of an acceptable scientific standard.

# Peer review: Reviewer response for version 1

**DOI:** 10.5256/f1000research.30253.r74624

**Published:** 2020-11-23

**Authors:** Natalia Szejko

**Affiliations:** 1 Department of Neurology, Medical University of Warsaw, Warsaw, Poland; 2 Department of Neurology, Yale School of Medicine, Yale University, New Haven, CT, USA

This article offers greatly appreciated year summary of the most important articles about TS published in 2019. The authors gave a detailed overview of studies dedicated to tics and divided their findings into thematic sections to give these results more organization. I also appreciate the conclusions presented as questions raised by the researchers the previous year. 

 However, I would recommend to change a language for more formal and also correct several "typos". For example, in the results section, the authors of the study are not mentioned in this sentence:  "1 present a stimulating argument based on data that demonstrate a severity continuum between chronic tics and TS using a database regrouping 1018 subjects"; the sentence is merely starting with the reference, instead. The same happens at several other occasions. In some points, I also missed more detailed conclusions, for example regarding the TBI risk in TS, where it is only stated that "These findings have important therapeutic implications". I would also introduce the separated section regarding the reviews of some particular topics, as in the "Pathophysiology" section where the study by Singer and Augustine should be described with more details and proceeded by the heading "Reviews".

Is the review written in accessible language?

Partly

Are all factual statements correct and adequately supported by citations?

Yes

Are the conclusions drawn appropriate in the context of the current research literature?

Yes

Is the topic of the review discussed comprehensively in the context of the current literature?

Yes

Reviewer Expertise:

Tourette syndrome, movement disorders, neuropsychiatry, cannabis based medicine in neurology and psychiatry, functional movement disorders

I confirm that I have read this submission and believe that I have an appropriate level of expertise to confirm that it is of an acceptable scientific standard, however I have significant reservations, as outlined above.

# Response

**Published:** 2020-11-23

**Authors:** Andreas Hartmann

**Affiliations:** Pitié-Salpêtrière Hospital, Paris, France

We thank the reviewer for their appreciation of our work. 

 Regarding formatting (i.e., changing author names by numbers), this was done by F1000 and we will check with them whether it can be changed. The idea, obviously, was not to be informal but simply to stick to the rules. We have also rechecked for typos.

 As requested, we have expanded on the paper regarding TBI and TS.

 As to the tow reviews by Singer, they are exhaustive and do not point out to single results, so it is impossible to give a major result or take home message: we simply wished to bring them to the attention of readers wishing to dwell into the broader subject of TS pathophysiology.
